# Diabetes and Thyroid Cancer Risk: Literature Review

**DOI:** 10.1155/2012/578285

**Published:** 2012-06-12

**Authors:** Shyang-Rong Shih, Wei-Yih Chiu, Tien-Chun Chang, Chin-Hsiao Tseng

**Affiliations:** ^1^Department of Internal Medicine, National Taiwan University Hospital, No. 7, Chung Shan South Road, Taipei 10002, Taiwan; ^2^National Taiwan University College of Medicine, Taipei, Taiwan

## Abstract

Diabetic patients have a higher risk of various types of cancer. However, whether diabetes may increase the risk of thyroid cancer has not been extensively studied. This paper reviews and summarizes the current literature studying the relationship between diabetes mellitus and thyroid cancer, and the possible mechanisms linking such an association. Epidemiologic studies showed significant or nonsignificant increases in thyroid cancer risk in diabetic women and nonsignificant increase or no change in thyroid cancer risk in diabetic men. A recent pooled analysis, including 5 prospective studies from the USA, showed that the summary hazard ratio (95% confidence interval) for women was 1.19 (0.84–1.69) and was 0.96 (0.65–1.42) for men. Therefore, the results are controversial and the association between diabetes and thyroid cancer is probably weak. Further studies are necessary to confirm their relationship. Proposed mechanisms for such a possible link between diabetes and thyroid cancer include elevated levels of thyroid-stimulating hormone, insulin, glucose and triglycerides, insulin resistance, obesity, vitamin D deficiency, and antidiabetic medications such as insulin or sulfonylureas.

## 1. Introduction

**Figure 1:**
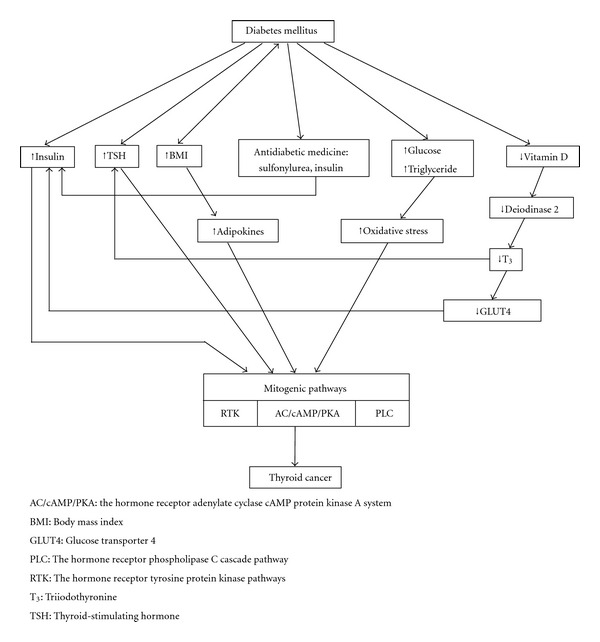
Pathophysiology proposed to link diabetes and thyroid cancer together. Diabetes mellitus may affect mitogenic pathway of the follicular cells through several mechanisms. Increased insulin amount stimulates follicular cells because of its structural similarity to insulin-like growth factor. Increased TSH stimulated AC/cAMP/PKA pathway. Increased body mass index will increase adipokines and subsequently stimulate mitogenic pathways. Antidiabetic medicines of sulfonylurea and insulin contribute to the elevated insulin level. Hyperglycemia and hypertriglycemia increase oxidative stress and stimulate mitogenic pathway. Vitamin D deficiency decreases deiodinase 2, T_3_ and GLUT4 transcription, which subsequently increase TSH and insulin levels and activate mitogenic pathways.

During the past several decades, the prevalence of diabetes has markedly increased [1–3]. Diabetes is associated with increased risk of various kinds of cancer, such as colon cancer, pancreatic cancer, breast cancer, bladder cancer, prostate cancer, and non-Hodgkin's lymphoma [4–8]. Meanwhile, the incidence of thyroid cancer is rising at a rate that is among the fastest of all malignancies [9]. According to a survey in the United States, the incidence of thyroid cancer increased by 2.4-fold from 1973 to 2002, and 87% of the increase consisted of cancers measuring 2 cm or smaller, but the mortality from thyroid cancer was stable [10]. It is believed that the major cause of this increase in incidence is the enhanced detection of early-stage tumors by the use of thyroid ultrasound and ultrasound-guided fine needle aspiration cytology examination. However, this cannot explain the increased prevalence preceding the widespread use of ultrasound [11]. It also cannot explain the increased incidence of large (>5 cm) papillary thyroid cancer [12]. Therefore, there may be some other contributing factors of the increased incidence of thyroid cancer. According to epidemiologic studies, exposure to ionizing radiation is the only clearly established risk factor [13]. Benign thyroid conditions and inadequate or excess iodine intake are the possible risk factors of thyroid cancer [13]. None of them can explain the increased thyroid cancer incidence. Statistic analysis showed that diabetes, obesity, and metabolic syndrome were potential risk factors of cancer development [14–16]. It is not clear whether diabetes plays a role in thyroid cancer risk. In this paper, we review the literature reporting the relationship between diabetes mellitus and thyroid cancer (summarized in Table 1) and the proposed mechanisms linking such an association (depicted in Figure 1).

## 2. Epidemiologic Findings

The prevalence of thyroid disorders among diabetics (10.8%) is higher than that in the general population (6.6%) [24]. Several studies disclosed the relationship between diabetes and thyroid cancer (Table 1) [13, 17, 18, 20–23]. Study number 8 [23] in Table 1 is a pooled analysis including 5 prospective studies from the USA, which included studies numbered 5 [13] and 7 [22]. Significant or nonsignificant increases in thyroid cancer risk were observed in diabetic women [13, 18, 19]. Nonsignificant increases or no change in thyroid cancer risk were observed in diabetic men [13, 17, 18, 20]. The results are controversial, and the link between diabetes and thyroid cancer is probably weak.

## 3. Hypotheses Proposed to Link Diabetes to Thyroid Cancer Risk

Currently, there are some hypotheses linking diabetes to thyroid cancer risk, including (i) increased body mass index (BMI); (ii) elevated insulin levels; (iii) long-term elevation of thyroid-stimulating hormone (TSH); (iv) long-term exposure to high levels of glucose and triglycerides; (v) vitamin D deficiency; (vi) use of antidiabetic medications including insulin and sulfonylureas [22] (Figure 1). There are some epidemiological studies demonstrating the relationships between thyroid cancer and BMI, TSH, blood glucose, and triglycerides.

### 3.1. Molecular Pathogenesis of Thyroid Cancer

In normal adults, the weight and composition of the thyroid glands remain generally constant [25]. The cells turnover about 6–8 renewals in adult life [25]. The cell growth is closely regulated by paracrine function of follicular cells, which secrete factors such as insulin-like growth factor I (IGF-1) and fibroblast growth factor to control other cells [26].

 In thyroid glands, three distinct mitogenic pathways have been proposed: (i) the hormone receptor adenylate cyclase-cAMP protein kinase A system (AC/cAMP/PKA); (ii) the hormone receptor tyrosine protein kinase (RTK) pathways; (iii) hormone receptor phospholipase C cascade (PLC) pathway [27]. TSH is the major stimulator of the AC/cAMP/PKA pathway by binding to the TSH receptor (TSHR). This pathway regulates the function, differentiation, and proliferation of the thyroid glands [28]. Epidermal growth factor (EGF) stimulates RTK pathway, which leads to an increase in transcriptional activity [29]. The PLC pathway is activated by TSH, neurotransmitters, growth factors, and phorbol ester. This pathway subsequently increases the intracellular calcium and protein kinase C activity [30]. There are two models of thyroid carcinogenesis: fetal cell carcinogenesis theory and multistep carcinogenesis theory [27]. Factors affecting the mitogenic pathways may be involved in the pathogenesis of thyroid cancer.

 Molecular pathogenesis involves genetic events [31]. Activating point mutations of the *RAS* genes is frequently found in follicular thyroid carcinomas [31]. Rearrangements of genes (*RET, TKR*) of transmembrane receptors with tyrosine kinase activity and activating point mutations of the *BRAF* gene are found in papillary thyroid carcinomas [31]. Poorly differentiated and anaplastic thyroid carcinomas are found to have inactivating point mutations of the *P53* gene [31]. Mutations of *RET* oncogene may also be responsible for the tumorigenesis of medullary thyroid cancer [31].

### 3.2. Elevated Insulin Levels and Thyroid Cancer Risk

Chronic elevated circulating insulin level is observed in diabetics and may be due to endogenous (insulin-resistance-related) or exogenous sources (medications). Insulin shares structural homology and affinity of the receptors with IGF-1, and is important for cell proliferation and apoptosis [32]. Elevated insulin and IGF-1 levels are related to various cancers, such as breast and colon cancers [33, 34]. As mentioned above, IGF-1 may control follicular cell growth [26]. In follicular cell cultures, incubation of follicular cells with TSH and insulin causes significant increase in cell number than incubation with TSH alone [35], suggesting that insulin may mimic IGF-1 in follicular cells. Follicular cells do synthesize IGF-1 and have IGF-1 receptors, which is associated with the pathogenesis of thyroid nodules by potentiating TSH action [36]. Therefore, insulin may also play a role in thyroid carcinogenesis. Some studies demonstrated the association between insulin resistance and thyroid nodules and thyroid cancer [37, 38]. However, to our knowledge, there has been no human study directly confirming the association between insulin exposure and thyroid cancer.

### 3.3. TSH and Thyroid Cancer Risk

As mentioned above, TSH is involved in mitogenic pathways of the thyroid glands [27]. TSH is an independent risk factor of thyroid cancer development [39–41]. Thyroid cancer risk increases with higher TSH level [40]. Higher TSH level is also associated with advanced stage of differentiated thyroid cancer [40]. Diabetic patients are more prone to have chronically mild TSH elevation. Previous study showed that 3% of insulin-dependent diabetics had hypothyroidism, and 13–20% had elevated TSH levels and antithyroid antibodies [42]. A recent study showed that the rate of primary hypothyroidism in type 2 diabetics is greater than in the nondiabetic population (odds ratio = 3.45; 95% CI: 2.51–4.79) [43]. The increased thyroid cancer risk may be related to the elevated TSH level in diabetic patients.

### 3.4. Increased BMI and Thyroid Cancer Risk

Obesity is associated with several types of cancer, such as adenocarcinoma of the esophagus, colon, kidney, endometrium, and malignant melanoma [44]. Obese people are at a 10-fold increased risk of diabetes [45]; and they may have increased risk of thyroid cancer [22, 46, 47]. Adjustment for BMI slightly reduced thyroid cancer risk associated with diabetes, but BMI only could not explain the association between diabetes and thyroid cancer [22]. Meta-analysis showed that an increase in BMI of 5 kg/m^2^ was associated with an increased risk of thyroid cancer in both men (RR = 1.33; *P* = 0.02) and women (RR = 1.14; *P* = 0.001) [47].

 Potential mechanisms linking obesity and thyroid cancer risk include elevated TSH levels, insulin resistance, and adipokines effect [9, 46]. Some studies showed that BMI and TSH levels were positively correlated, but others did not [46]. As mentioned above, TSH and insulin influence the growth and differentiation of follicular cells [27]. Adipokines such as adiponectin, leptin, and hepatocyte growth factor may regulate cancer cell proliferation and may be related to cancer progression [9]. Increased expression of leptin and its receptor in thyroid cancer were reported [48]. Its association with tumor aggressiveness and biological behavior was also demonstrated [48]. However, an inverse association was identified between BMI and tumor invasion and nodal metastasis in a clinicopathological cohort study [9]. Further study is necessary to determine the relationship between BMI and thyroid cancer outcome.

### 3.5. Antidiabetic Medications and Thyroid Cancer Risk

According to previous studies, cancer risk in metformin-treated patients is similar to that in patients not receiving medication for diabetes [49]. Metformin diminishes growth stimulation by insulin and inhibits growth of thyroid cancer *in vitro* [50]. There are several mechanisms proposed for the antitumor effect of metformin such as increasing the AMP-activated protein kinase signaling pathway and a direct influence upon immune competence [51].

Sulfonylureas are associated with increased mortality (HR = 1.3; 95% CI: 1.1–1.6) [52]. Cancer mortality is about doubled among insulin users relative to metformin users (HR = 1.9; 95% CI: 1.5–2.4) [52]. Cancer risk increases by an estimated 20% for each year of insulin therapy [53]. Increased circulating insulin level may be another explanation for the increased cancer risk associated with sulfonylureas and insulin therapy [51]. As mentioned above, high insulin levels and the associated changes of the IGF-1 axis may be associated with cancer development. Glargine, a long-acting insulin analog, may have even higher cancer risks compared with human insulin [51]. This is possibly due to the prolonged binding of IGF-1 receptor, leading to increased mitotic activity [51]. To our knowledge, there has been no human study confirming the association between insulin and sulfonylurea treatment and thyroid cancer [22].

 The association of incretin-based therapy and medullary thyroid cancer had been widely discussed. Glucagon-like pepide-1 receptor activation promotes C-cell proliferation and medullary thyroid cancer in rodents [54, 55]. Currently, there is no sufficient data to confirm the association between incretin-based therapies and thyroid cancer in humans [56].

Other potential drugs are peroxisome proliferator-activated receptors (PPARs) *γ* agonists, which has been demonstrated to promote the growth and invasion of thyroid cancer cells* in vitro *with an increase in G1 phase and a decrease in the S and G2/M phases [57]. But the mechanism is unclear.

### 3.6. Chronic Glucose and Triglycerides Exposure and Thyroid Cancer Risk

Studies showed that men with elevated level of triglycerides and women with increased blood glucose level were more prone to have thyroid cancer [14, 58, 59]. The possible mechanism is the increased oxidative stress. Free fatty acids and glucose stimulate nuclear factor-*κ* B, which increases the production of nitric oxide, a substrate for reactive oxygen species (ROS) [60]. Low level of ROS regulates cellular signaling and is important in normal cell proliferation. Increased ROS is observed in cancer cells [14]. However, a recent large-scale cohort analysis shows that glucose was inversely associated with thyroid cancer risk in women below 50 years old, was not related to thyroid cancer risk in women above 50 years old, and was associated with an increased thyroid cancer risk in men [61]. The proposed mechanism is the complex relationship among reproductive hormones, glucose, and thyroid cancer. Since the study lacks detailed information on reproductive history and sex hormone use, it cannot come to any conclusion. In summary, current human studies showed controversial relationship between glucose level and thyroid cancer risk.

### 3.7. Vitamin D Deficiency and Thyroid Cancer Risk

Vitamin D deficiency is observed in up to 70% of diabetics, although the reason is unclear [22, 62]. Vitamin D promotes differentiation and apoptosis of cancer cells [63]. Low vitamin D level decreases deiodinase 2, resulting in decreased intracellular triiodothyronine (T_3_) [22]. Decreased T_3_ concentration in skeletal muscle and adipose tissue should lead to decreased glucose transporter 4 transcription and thus lead to insulin resistance [22]. Decreased T_3_ concentration in pituitary gland stimulates TSH release [22]. As mentioned above, insulin resistance and TSH may be related to thyroid cancer. To our knowledge, there has been no human study directly confirming the association between vitamin D deficiency and thyroid cancer.

## 4. Conclusion

Epidemiologic studies showed significant or nonsignificant increases in thyroid cancer risk in diabetic women, and nonsignificant increases or no change in thyroid cancer risk in diabetic men. The results are controversial, and evidence is not strong enough to link diabetes and thyroid cancer. Mechanisms proposed to link diabetes and thyroid cancer include elevated TSH, insulin, glucose, triglycerides, insulin resistance, obesity, vitamin D deficiency, and antidiabetic medications. However, these mechanisms are mostly postulated from epidemiological studies, and studies providing direct biological modes of action are still scarce. Further research is necessary to confirm the relationship between diabetes and thyroid cancer and to explore the underlying mechanisms.

## Figures and Tables

**Table 1:** Summary of available studies evaluating the relationships between diabetes and thyroid cancer.

Study no. [reference]	Year author	Country	Name of study	Study design	Number of cases/ Follow-up duration	Estimated risk
1 [17]	1991 Adami et al.	Sweden	Cancer risk in patients with diabetes mellitus	Population-based cohort study	51,008 patients. Cohort established by identifying diabetic patients during 1965–1983. Complete followup through 1984	Women: RR = 1 (95% CI: 0.6–1.8) Men: RR = 1.3 (95% CI: 0.5–2.8)

2 [18]	1997 Wideroff et al.	Denmark	Cancer incidence in a population-based cohort of patients hospitalized with diabetes mellitus in Denmark	Prospective cohort study	109,581 diabetics. Cohort established by identifying diabetic patients during 1977–1989. Cohort exit date: date of death or 1993	Women: SIR = 1.3 (95% CI: 0.6–2.3) Men: SIR = 1.2 (95% CI: 0.7–1.8)

3 [19]	2006 Inoue et al.	Japan	The Japan Public Health Center-based Prospective Study	Prospective cohort study	46,548 women, 51,223 men Followed from 1990 through 2003	Women: HR = 1.11 (95% CI: 0.35–3.5) Men: NA

4 [20]	2007 Kuriki et al.	Japan	Hospital based Epidemiologic Research Program at Aichi Cancer Center, Japan	Case-control study	11,672 incident cancer cases (5341 men, 6331 women) 47,768 cancer-free controls (14,199 men, 33,569 women)	Women: OR = 0.67 (95% CI: 0.21–2.10) Men: OR = 1.07 (95% CI: 0.33–3.48)

5 [13]	2010 Meinhold et al.	USA	The US Radiologic Technologists Study	Prospective cohort study	69,506 women, 21,207 men Followed from 1983 through 2006	Women: HR = 1.37 (95% CI: 0.49–3.77) Men: NA

6 [21]	2010 Chodick et al.	Israel	Diabetes and risk of incident cancer: a large population-based cohort study in Israel	Retrospective cohort study	16,721 DM, 83,874 non-DM Mean follow-up time: 8 years	Women: HR = 1.61 (95% CI: 0.96–2.69) Men: HR = 0.72 (95% CI: 0.25–2.04)

7 [22]	2011 Aschebrook-Kilfoy et al.	USA	The NIH-AARP Diet and Health Study	Prospective cohort study	200,556 women, 295,992 men Mean follow-up time: 10 years	Women: HR = 1.54 (95% CI: 1.08–2.20) Men: HR = 1.11 (95% CI: 0.74–1.66)

8 [23]	2012 Kitahara et al.	USA	Physical activity, diabetes, and thyroid cancer risk: a pooled analysis of five prospective studies	Pooled analysis of five prospective studies, including NIHAARP Diet and Health Study (NIH-AARP), Prostate, Lung, Colorectal, and Ovarian Cancer Screening Trial (PLCO), Breast Cancer Detection and Demonstration Project (BCDDP), Agricultural Health Study (AHS), and US Radiologic Technologists Study (USRT)	312,149 women, 362,342 men Median follow-up time: 10.5 years	Women: HR = 1.19 (95% CI: 0.84–1.69) Men: HR = 0.96 (95% CI: 0.65–1.42)

Studies no. 5 and 7 were included in the pooled analysis of study 8. CI: confidence interval, HR: Hazard ratio, OR: odds ratio, RR: relative risk, SIR: site-specific standardized incidence ratio, NA: not available.
